# Bladder urothelium converts bacterial lipopolysaccharide information into neural signaling via an ATP-mediated pathway to enhance the micturition reflex for rapid defense

**DOI:** 10.1038/s41598-020-78398-9

**Published:** 2020-12-03

**Authors:** Norichika Ueda, Makoto Kondo, Kentaro Takezawa, Hiroshi Kiuchi, Yosuke Sekii, Yusuke Inagaki, Tetsuji Soda, Shinichiro Fukuhara, Kazutoshi Fujita, Motohide Uemura, Ryoichi Imamura, Yasushi Miyagawa, Norio Nonomura, Shoichi Shimada

**Affiliations:** 1 Department of Neuroscience and Cell Biology, Osaka University Graduate School of Medicine, 2-2 Yamada-oka, Suita, Osaka, 565-0871 Japan; 2 Department of Urology, Osaka University Graduate School of Medicine, Osaka, 565-0871 Japan; 3 Addiction Research Unit, Osaka Psychiatric Research Center, Osaka Psychiatric Medical Center, Osaka, 541-8567 Japan

**Keywords:** Urology, Bladder

## Abstract

When bacteria enter the bladder lumen, a first-stage active defensive mechanism flushes them out. Although urinary frequency induced by bacterial cystitis is a well-known defensive response against bacteria, the underlying mechanism remains unclear. In this study, using a mouse model of acute bacterial cystitis, we demonstrate that the bladder urothelium senses luminal extracellular bacterial lipopolysaccharide (LPS) through Toll-like receptor 4 and releases the transmitter ATP. Moreover, analysis of purinergic P2X_2_ and P2X_3_ receptor-deficient mice indicated that ATP signaling plays a pivotal role in the LPS-induced activation of L6–S1 spinal neurons through the bladder afferent pathway, resulting in rapid onset of the enhanced micturition reflex. Thus, we revealed a novel defensive mechanism against bacterial infection via an epithelial-neural interaction that induces urinary frequency prior to bacterial clearance by neutrophils of the innate immune system. Our results indicate an important defense role for the bladder urothelium as a chemical-neural transducer, converting bacterial LPS information into neural signaling via an ATP-mediated pathway, with bladder urothelial cells acting as sensory receptor cells.

## Introduction

The urinary bladder is an organ leading to the outside of the body. The urethral opening is directly connected to the urinary bladder and is in close proximity to the gastrointestinal tract and vagina mucosa where numerous bacteria reside^1,2^. Therefore, the urinary bladder is routinely exposed to bacteria that retrogradely enter from the outside^3^. Although urine can serve as a bacterial growth medium^4^, the urinary bladder normally remains uninfected because it possesses inherent defensive mechanisms. The luminal surface of the urinary bladder is lined by the bladder urothelium, which is stratified and comprised of three cell types, umbrella cells, intermediate cells, and basal cells^5^. The umbrella cells interface with urine and form a highly resistant physical barrier that restricts the invasion of bacteria into the body^5–7^. The barrier is maintained by tight junctions between umbrella cells and uroplakins located on the apical surface of umbrella cells^3,7^. In addition, the polysaccharides, glycosaminoglycan and mucin, cover the superficial umbrella cells and prevent bacterial attachment^5,7,8^. However, the physical barrier of the urothelium alone is insufficient to defend against bacterial infection. Therefore, more active defensive mechanisms are needed.

The innate immune response of the bladder is an essential active defensive mechanism against bacterial infection^3,9–11^. That is, the bladder urothelium and resident leukocytes sense bacterial activity and promote the expression of inflammatory cytokines and chemokines, resulting in neutrophil influx into the bladder tissue and lumen^3,9–11^. These neutrophils subsequently phagocytose bacteria in the bladder. This neutrophil response has been widely recognized as the central defensive mechanism against bacterial cystitis^11^.

In addition to its barrier function, the bladder urothelium also has sensory and signaling functions, which has recently attracted much interest^5,7,12–14^. The bladder urothelium has numerous receptors and ion channels and releases a number of neurotransmitters and mediators^5,7,12,14,15^. A dense sensory nerve network has been identified in the suburothelial layer of the bladder, with some terminal fibers projecting into the urothelium and others terminating between muscles^16,17^. Transmitters released from the urothelium regulate the activity of the bladder afferent nerves^5,12,18^, and play an important role in the transduction of bladder stimuli to the central nervous system^7,17,19^. Among the transmitters released from the urothelium, ATP is considered to play a key role in bladder sensory functions^5,7,20^. ATP is released from the bladder urothelium in response to mechanical and chemical stimuli^21–24^, and purinergic receptors are expressed on a subset of bladder afferent nerves^5,20,25,26^. ATP can act on bladder afferent nerves via purinergic receptors to regulate bladder function^20,25,27,28^. Additionally, the involvement of altered ATP signals in functional bladder disorders, such as intestinal cystitis and overactive bladder, has been suggested^29–31^. Thus, accumulating evidence demonstrates the significance of urothelial ATP signaling via purinergic receptors in bladder function^32,33^.

Urinary frequency is a common symptom of bacterial cystitis and is a well-known defensive response against bacteria^2^. Because bacteria in the bladder cavity can be pushed out rapidly, frequent urination appears to be an effective response to defend against bacterial infection^34,35^. However, the underlying mechanism of urinary frequency in bacterial cystitis remains unclear. Furthermore, although ATP has been suggested to modulate urinary function in bacterial infection^36^, the precise process is not well understood. In this study, using a mouse model of acute bacterial cystitis induced by lipopolysaccharide^37,38^, we investigated bladder ATP signaling during bacterial cystitis, and we aimed to clarify the mechanism underlying the expulsion of bacteria from the bladder.

## Results

### Lipopolysaccharide induces urinary frequency more rapidly than concomitant inflammatory changes in the bladder and urine

**Figure 1:**
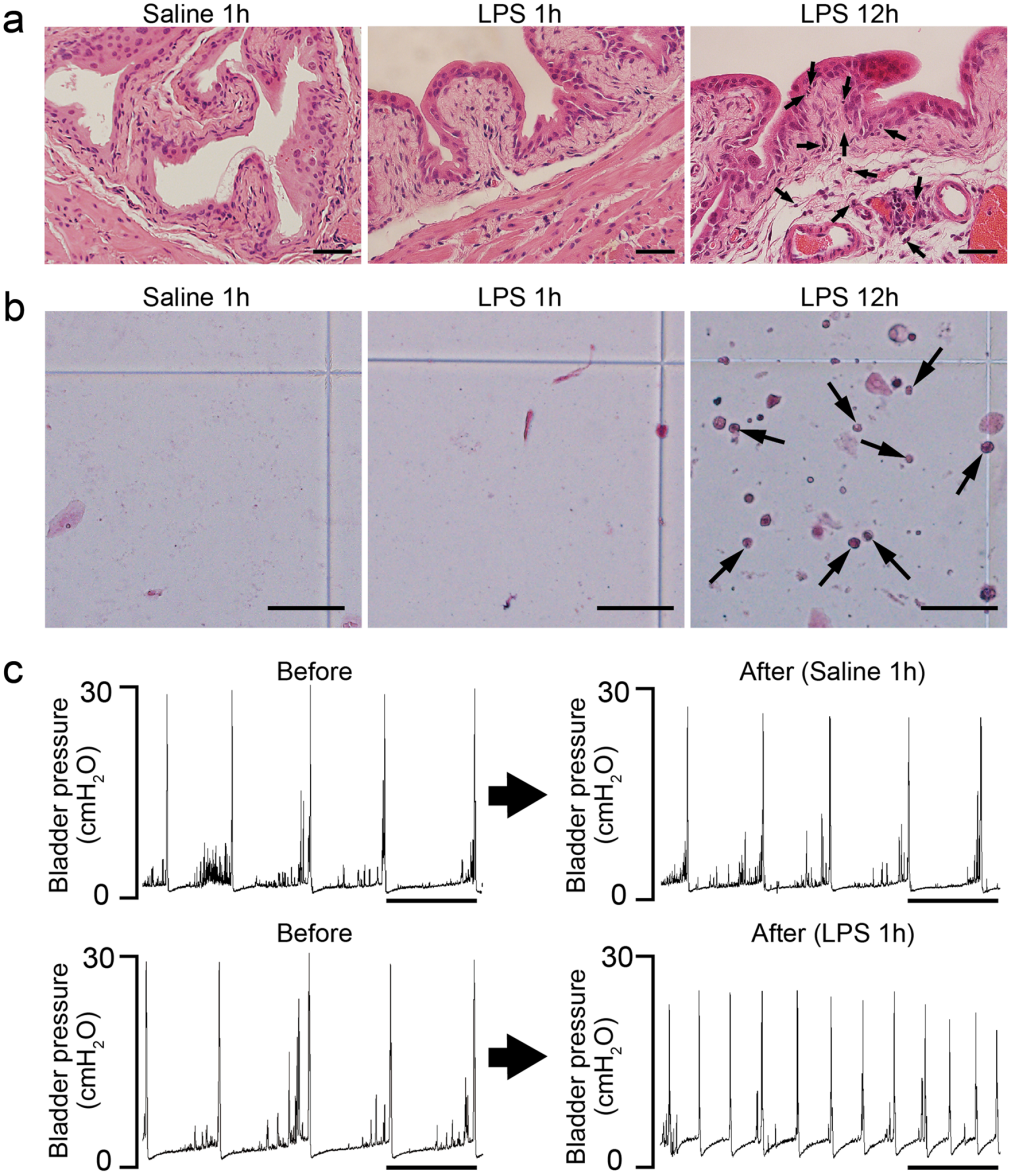
LPS induces urinary frequency more rapidly than inflammatory changes in the bladder and urine. (**a**) Representative images of the bladder stained with hematoxylin and eosin after intravesical exposure to saline or LPS. Images at 1 h after administration of saline (left) or LPS (middle) and 12 h after LPS administration (right) are shown. Arrows indicate neutrophils. Scale bars, 40 μm. (**b**) Representative images of Samson stained urine after intravesical exposure to saline or LPS. Images at 1 h after administration of saline (left) or LPS (middle), and 12 h after LPS administration (right) are shown. Arrows indicate neutrophils. Scale bars, 50 μm. (**c**) Representative cystometrograms of saline and LPS instillation in wild-type mice. Scale bars, 10 min. At least three independent experiments were performed and similar results were obtained.

Lipopolysaccharide (LPS) is the major component of the Gram-negative bacterial cell wall and a common virulence factor^38^. LPS has been widely used in experimental animal models of acute bacterial cystitis^37,38^. Twelve hours after exposure of the bladder lumen to LPS, inflammatory histological changes developed in the bladder, characterized by neutrophil infiltration, interstitial edema, vasodilation, and microhemorrhages (Fig. 1a right), as previously reported^39^. In addition, neutrophils were detected in urine at 12 h after LPS exposure (Fig. 1b right). Interestingly, functional analysis of the bladder by cystometry within 1 h after intravesical LPS exposure revealed urinary frequency [i.e. decreased intercontraction intervals (ICIs)] (Fig. 1c), although there were no apparent inflammatory changes in the bladder or urine (Fig. 1a middle, b middle). These results indicate that LPS induces urinary frequency more rapidly than the concomitant inflammatory changes in the bladder and urine.

### Intravesical LPS instillation induces urinary frequency through Toll-like receptor 4

**Figure 2:**
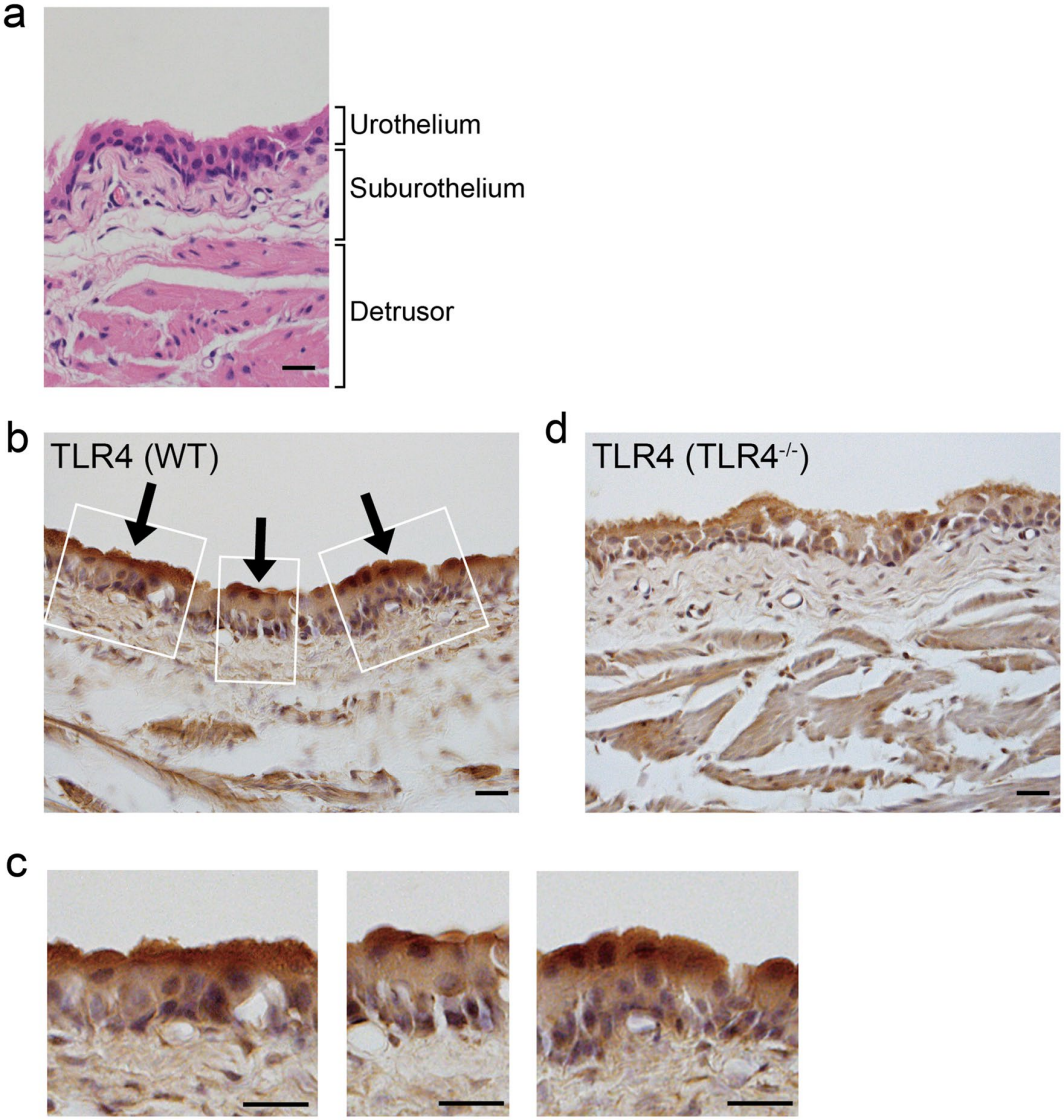
Specific expression pattern of TLR4 in the bladder urothelium. (**a**) Representative image of wild-type bladder stained with hematoxylin and eosin. (**b**–**d**) Immunohistochemical staining of TLR4 with hematoxylin counter-staining in the bladder of wild-type mice (**b**, **c**) and TLR4^–/–^ mice (**d**). Panels in (**c**) show magnified views of boxed areas in (**b**). Arrows indicate TLR4 staining. Scale bars, 20 μm. At least three independent experiments were performed and similar results were obtained.

LPS is known to act through Toll-like receptor 4 (TLR4)^38^. To reveal the mechanism of urinary frequency induced by intravesical LPS instillation, we first investigated the TLR4 expression pattern in the bladder. To examine TLR4 expression in the urothelium, we performed immunohistochemical analysis combined with hematoxylin staining. Interestingly, strong immunoreactivity for TLR4 was observed on the apical side of the umbrella urothelium (Fig. 2a–d), which corroborates a previous report^40^. A specific expression pattern of TLR4 in the umbrella urothelium is indicative of its functional role against luminal bacterial LPS.

**Figure 3:**
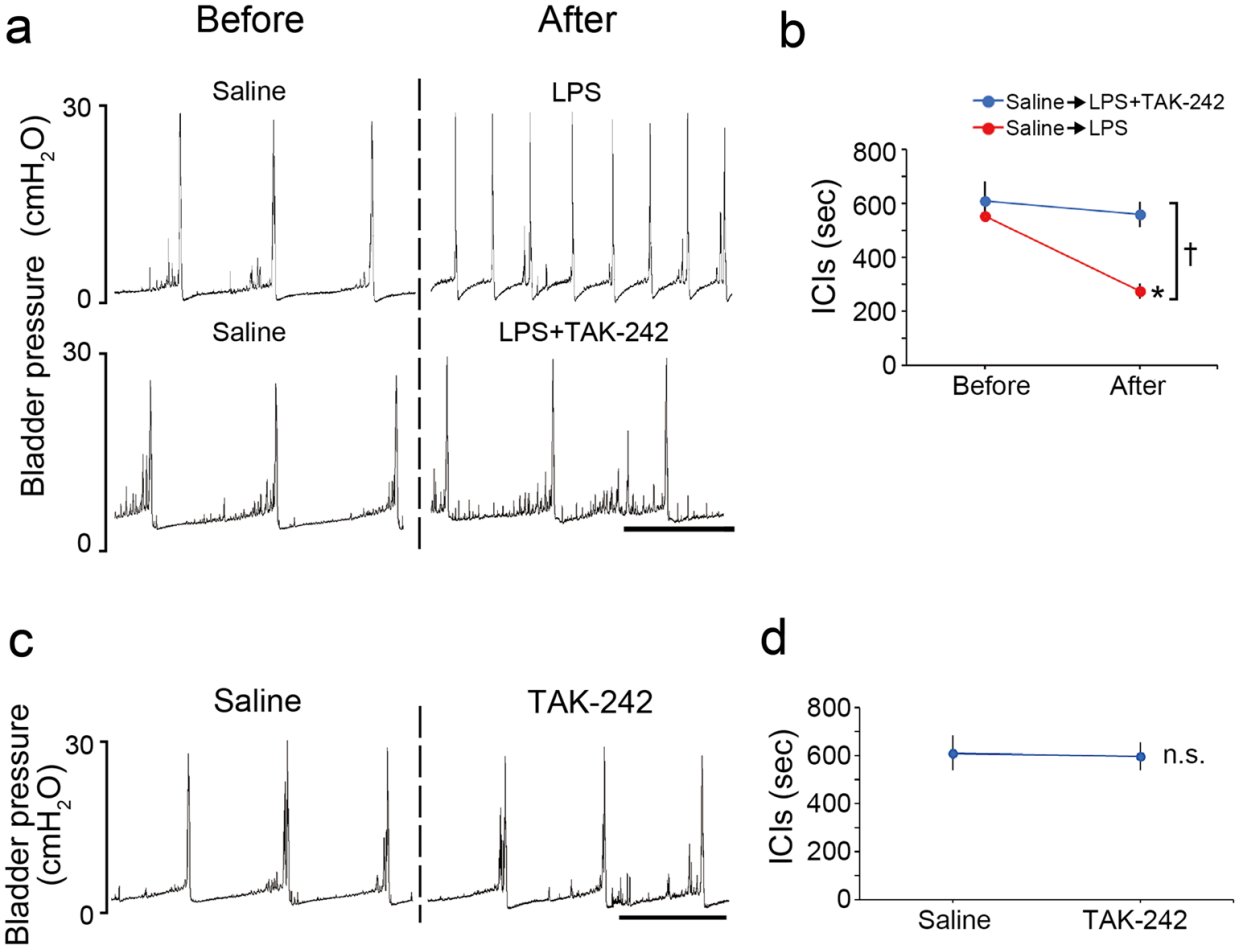
Intravesical LPS instillation induces urinary frequency through TLR4. (**a**) Representative cystometrograms after LPS instillation with or without TAK-242 treatment in wild-type mice. Scale bar, 10 min. (**b**) Changes in ICIs after LPS instillation with or without TAK-242 treatment (*n* = 3 mice per group); *p = 0.023 (paired *t*-test with Holm correction) and ^†^*p* = 0.003 (two-way repeated measures ANOVA). (**c**) Representative cystometrograms after TAK-242 treatment in wild-type mice. Scale bar, 10 min. (**d**) Changes in ICIs after TAK-242 treatment (*n* = 3 mice); *p* = 0.26 (paired *t*-test). Error bars represent s.e.m., n.s., not significant.

We next examined the possible role of TLR4 in the LPS-induced urinary frequency. Cystometry revealed that intravesical instillation of a TLR4 selective antagonist, TAK-242, attenuated the LPS-induced urinary frequency (Fig. 3a,b) (*p = 0.023, paired *t*-test with Holm correction; ^†^*p* = 0.003, two-way repeated measures ANOVA). Meanwhile, treatment with TAK-242 alone did not affect ICIs (Fig. 3c, d) (*p* = 0.26, paired *t*-test). These results indicate that urinary frequency induced by luminal bacterial LPS is mediated by TLR4 in the urothelium.

### LPS acts on TLR4 and induces ATP release from the urothelium

**Figure 4:**
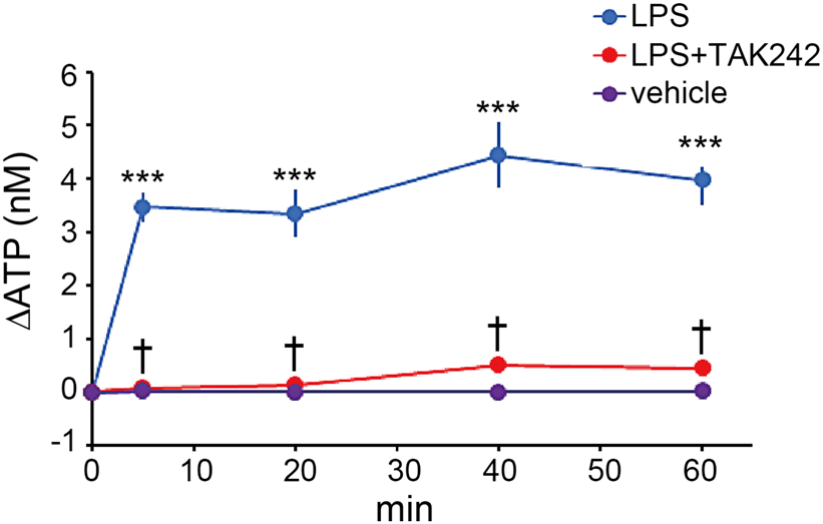
LPS acts on TLR4 and induces ATP release from the urothelium. An ATP release assay showing the time course of ATP concentration (ΔATP) in the chamber solution after LPS treatment with or without TAK-242 (*n* = 4 mice per group); ***p < 0.001 versus vehicle, and ^†^*p* < 0.001 versus LPS (Tukey’s test following one-way ANOVA with Holm correction). Error bars represent s.e.m.

ATP is a signaling molecule that regulates diverse cellular processes^20^. Specifically, ATP is released from the urothelium and is important in bladder function^7,25,32,33^. Thus, we first examined ATP release from the urothelium in response to LPS treatment using an ATP release assay in isolated bladders^24^. LPS treatment induced rapid ATP release towards the mucosal side (Fig. 4), as previously reported^23,24^ (***p < 0.001, Tukey’s test following one-way ANOVA with Holm correction). Importantly, this ATP release was entirely blocked by TAK-242 treatment (Fig. 4) (^†^*p* < 0.001, Tukey’s test following one-way ANOVA with Holm correction). There were no significant differences in the pre-stimulation ATP concentration among groups (Supplementary Fig. S1) (*p* = 0.50, one-way ANOVA). These results indicate that LPS acts directly on TLR4 and induces ATP release from the urothelium.

### ATP signaling plays a pivotal role in LPS-induced activation of L6–S1 spinal neurons

Bladder primary afferent nerves transmit information from the bladder to neurons in the spinal cord^17^. The L6–S1 spinal neurons receive most of this afferent input from the bladder and are involved in its processing^41–43^. In the dorsal horn of the spinal cord, excitatory neural afferent input generates rapid trans-synaptic expression of c-Fos, which is a marker of neuron activation^41–45^. Thus, to examine neuronal activity following intravesical LPS stimulation, we performed immunohistochemical analysis of c-Fos in L6–S1 spinal neurons. Intravesical LPS instillation significantly increased the number of c-Fos-positive cells in the L6–S1 spinal cord in wild-type mice (Supplementary Fig. S2a), as was previously observed for other chemical substances^41,42^. This indicates that luminal LPS induces activation of L6–S1 spinal neurons through the bladder afferent nerves. Detailed analysis^41,42,45^ revealed that the increase in the number of c-Fos-positive cells was mainly localized to regions of the dorsal commissure (DCM) and the sacral parasympathetic nucleus (SPN) of the L6–S1 spinal cord (Supplementary Fig. S2b,c) (*p = 0.028, Student’s *t*-test with Holm correction).

**Figure 5:**
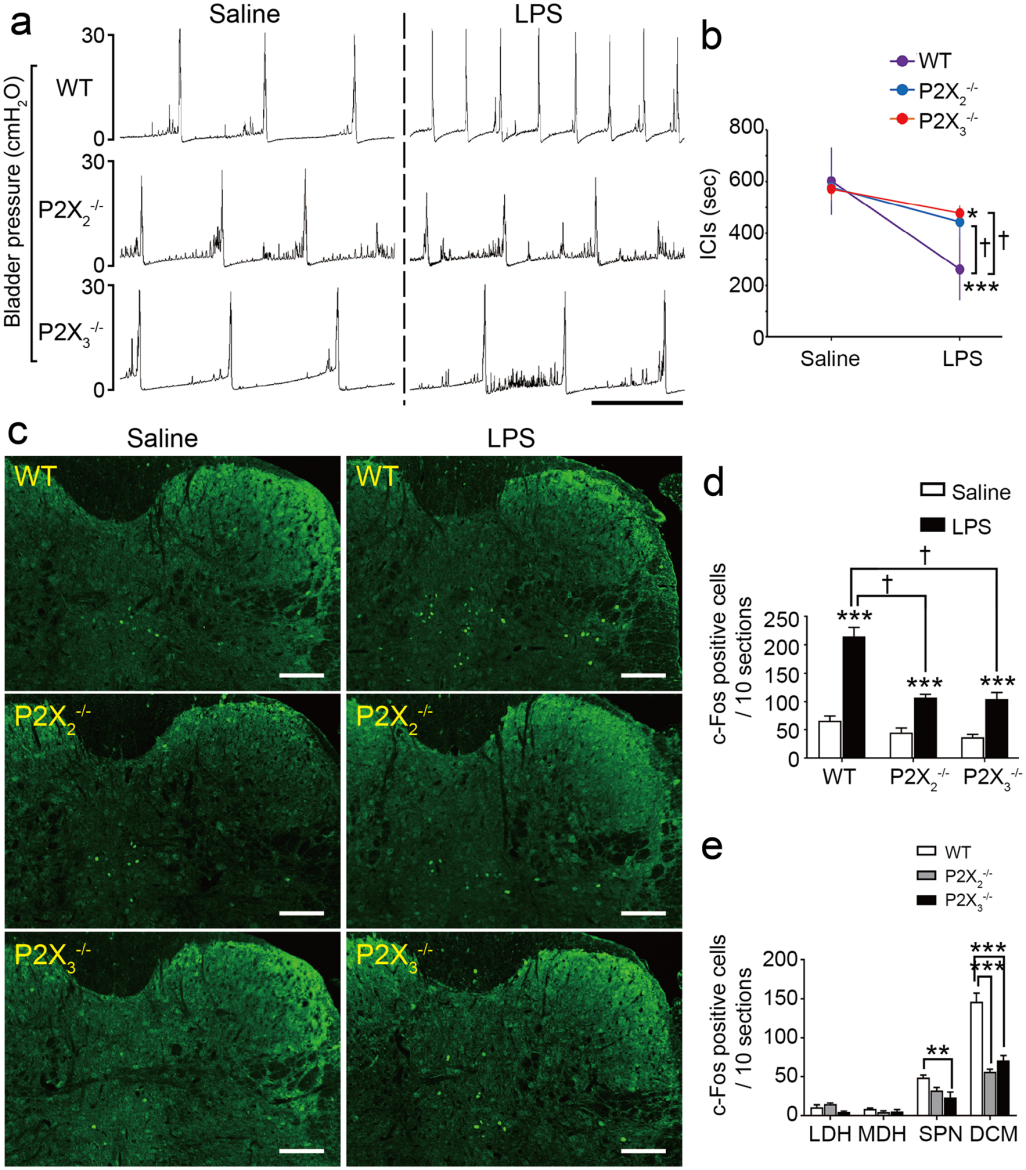
ATP signaling via P2X_2_ and P2X_3_ receptors plays an important role in the LPS-induced activation of L6–S1 spinal neurons. (**a**) Representative cystometrograms after LPS instillation in wild-type, P2X_2_^–/–^, and P2X_3_^–/–^ mice. Scale bar, 10 min. (**b**) Changes in ICIs after LPS treatment (*n* = 4 mice per group); *p = 0.018 in P2X_3_^–/–^ mice and ***p < 0.001 in wild-type mice (paired *t*-test with Holm correction). ^†^*p* = 0.016 and ^†^*p* < 0.001 in wild-type versus P2X_2_^–/–^ and P2X_3_^–/–^ mice, respectively (time by group interaction using two-way repeated measures ANOVA with Holm correction). (**c**) Immunohistochemical analysis of c-Fos expression in L6–S1 spinal cord after saline or LPS instillation in wild-type, P2X_2_^–/–^, and P2X_3_^–/–^ mice. Scale bars, 100 μm. (**d**) LPS-induced increases in c-Fos-positive cell numbers in L6–S1 spinal cord of wild-type, P2X_2_^–/–^, and P2X_3_^–/–^ mice (*n* = 5 mice per group: 10 sections per mouse were assessed); ***p < 0.001 (Student’s *t*-test with Holm correction); ^†^*p* = 0.0025 in wild-type versus P2X_2_^–/–^ mice and ^†^*p* = 0.0046 in wild-type versus P2X_3_^–/–^ mice (LPS by group interaction using two-way repeated measures ANOVA with Holm correction). (**e**) The distribution of c-Fos-positive cells in the L6–S1 spinal cord induced by LPS instillation in wild-type, P2X_2_^–/–^, and P2X_3_^–/–^ mice (*n* = 5 mice per group: 10 sections per mouse were assessed); **p = 0.0089 in wild-type versus P2X_3_^–/–^ mice in SPN, and ***p < 0.001 in wild-type versus P2X_2_^–/–^ and P2X_3_^–/–^ mice in DCM (Tukey’s test following one-way ANOVA). Error bars represent s.e.m.

We next investigated the possible role of ATP signaling in LPS-induced neuronal activation in the spinal cord. Purinergic P2X_2_ and P2X_3_ receptors are expressed on a subset of bladder afferent nerves^25,26^. In mice, the majority of lumbosacral dorsal root ganglion neurons, which constitute the primary afferent nerves, express P2X_2_, P2X_3_ and/or P2X_2/3_ receptors^14,46^. These purinergic receptors are also expressed in urothelial cells^5,47^. Our recent detailed analysis of mouse bladder function showed that LPS-induced urinary frequency was attenuated in P2X_2_^–/–^ and P2X_3_^–/–^ mice^24^. The cystometrogram recordings (Fig. 5a) and quantification of ICI changes (Fig. 5b) (P2X_2_^–/–^ mice, ^†^*p* = 0.016; P2X_3_^–/–^ mice, ^†^*p* < 0.001, time by group interaction using two-way repeated measures ANOVA with Holm correction) corroborate our recent reported findings^24^. Therefore, to examine the role of ATP signaling in the LPS-induced activation of L6–S1 spinal neurons, we performed c-Fos expression analysis with P2X_2_^–/–^ and P2X_3_^–/–^ mice following intravesical LPS instillation. Importantly, the increase in the number of LPS-induced c-Fos-positive cells in the L6–S1 spinal cord was significantly lower in both P2X_2_^–/–^ and P2X_3_^–/–^ mice compared with that in wild-type mice (Fig. 5c,d) (P2X_2_^–/–^ mice, ^†^*p* = 0.0025; P2X_3_^–/–^ mice, ^†^*p* = 0.0046, LPS by group interaction using two-way repeated measures ANOVA with Holm correction). The c-Fos-positive cell number was primarily suppressed in the DCM region in P2X_2_^–/–^ and P2X_3_^–/–^ mice (Fig. 5e) (***p < 0.001, Tukey’s test following one-way ANOVA). These results indicate that ATP signaling via P2X_2_ and P2X_3_ receptors plays a pivotal role in the LPS-induced activation of L6–S1 spinal neurons and the resulting enhanced micturition reflex.

### Intravesical ATP treatment triggers activation of L6–S1 spinal neurons and enhances the micturition reflex via purinergic receptors

**Figure 6:**
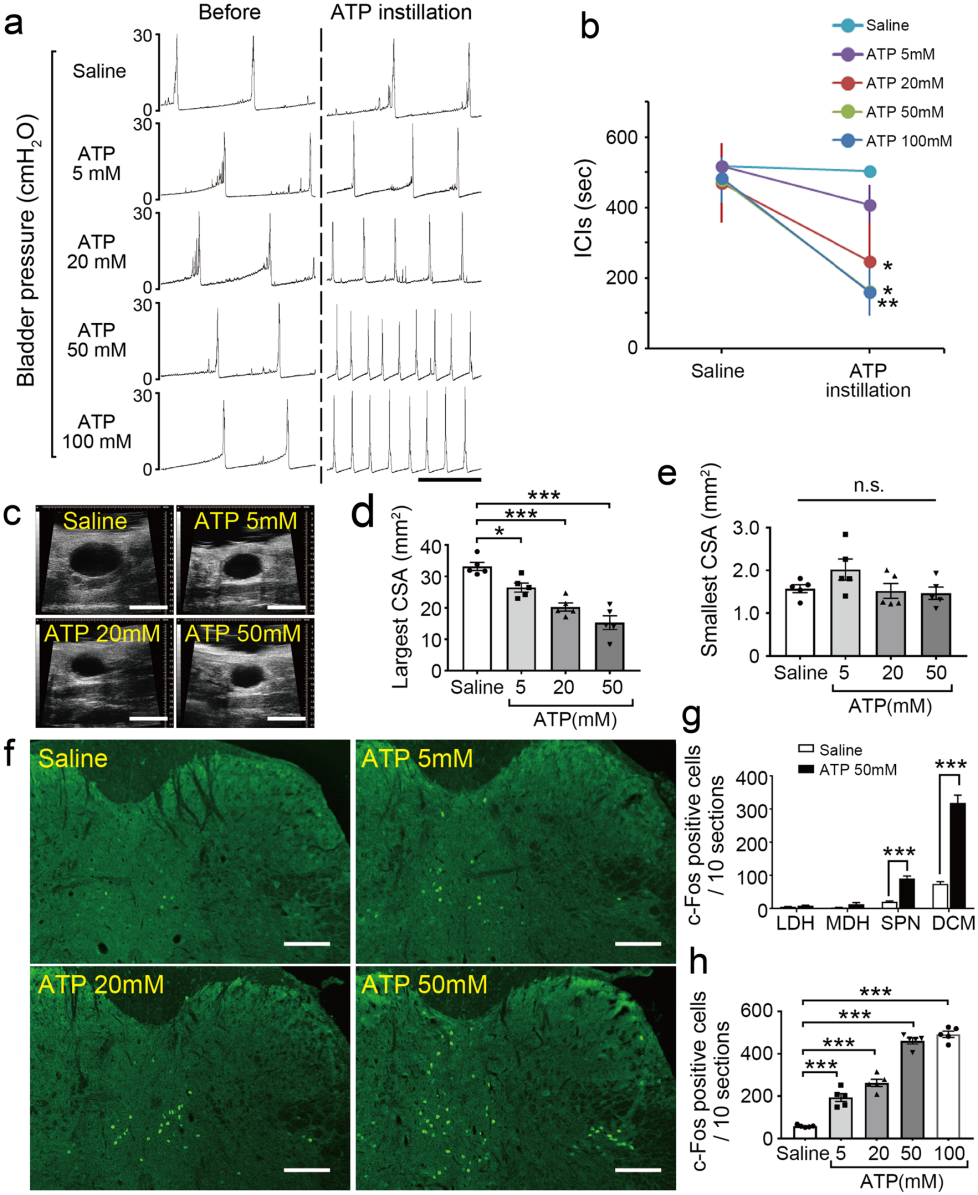
Intravesical ATP treatment triggers activation of L6–S1 spinal neurons and induces an enhanced micturition reflex. (**a**) Representative cystometrograms after ATP instillation in wild-type mice. Scale bar, 10 min. (**b**) Changes in ICIs after ATP treatment (*n* = 5 mice per group); *p = 0.041 for 20 mM ATP, *p = 0.010 for 50 mM ATP, and **p = 0.0018 for 100 mM ATP (paired *t*-test with Holm correction). (**c**) Ultrasonographic findings of pre-voiding with intravesical ATP treatment. Scale bars, 5 mm. (**d**, **e**) Changes in largest CSA (**d**) and smallest CSA, post-voiding (**e**) (*n* = 5 mice per group); *p = 0.041 in saline versus 5 mM ATP, and ***p < 0.001 in saline versus 20 mM ATP and 50 mM ATP (Tukey’s test following one-way ANOVA). (**f**) Immunohistochemical analysis of c-Fos expression in L6–S1 spinal cord after ATP instillation. Scale bars, 100 μm. (**g**) The distribution of c-Fos-positive cells in the L6–S1 spinal cord induced by ATP instillation (50 mM) in wild-type mice (*n* = 5 mice per group); ***p < 0.001 in SPN and DCM (Student’s *t*-test with Holm correction). (**h**) The number of c-Fos-positive cells in L6–S1 spinal cord after ATP instillation (*n* = 5 mice per group: 10 sections per mouse were assessed). ***p < 0.001 in saline versus all ATP concentrations (Tukey’s test following one-way ANOVA). Error bars represent s.e.m., n.s., not significant.

To further study the role of ATP signaling in bladder function, we examined the effects of intravesical ATP treatment on the micturition reflex. Cystometry of wild-type mice showed that intravesical ATP instillation decreased ICIs in a dose-dependent manner (Fig. 6a,b) (20 mM, *p = 0.041; 50 mM, *p = 0.010; 100 mM, **p = 0.0018, paired *t*-test with Holm correction). Ultrasonographic images, which enable more detailed examination of bladder function^48^, showed that ATP instillation in wild-type mice decreased the largest cross-sectional area (CSA) of the bladder in a dose-dependent manner (Fig. 6c,d) (5 mM, *p = 0.041; 20 mM, ***p < 0.001; 50 mM, ***p < 0.001, Tukey’s test following one-way ANOVA). Meanwhile, the smallest CSA was not affected after ATP instillation (Fig. 6e) (5 mM, *p* = 0.29; 20 mM, *p* = 1,00; 50 mM, *p* = 0.97). Furthermore, intravesical ATP instillation in wild-type mice induced c-Fos expression in the L6–S1 spinal cord with a similar distribution to that induced by LPS instillation (Fig. 6f,g, Supplementary Fig. S3) (***p < 0.001, Student’s *t*-test with Holm correction), and increased the number of c-Fos-positive cells in a dose-dependent manner (Fig. 6f,h, Supplementary Fig. S3) (***p < 0.001, Tukey’s test following one-way ANOVA). These results in wild-type mice are consistent with our previous report^43^.

**Figure 7:**
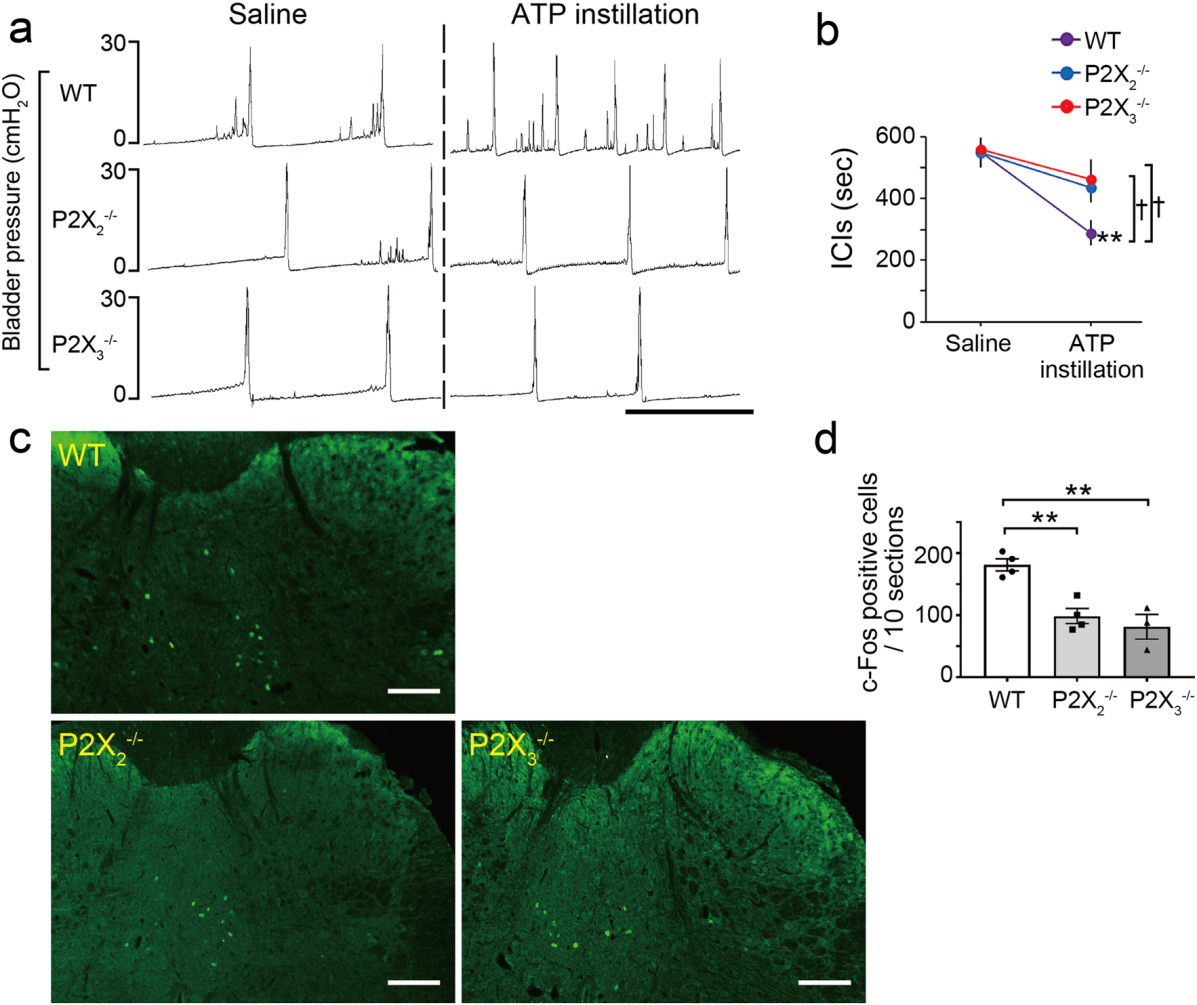
Effects of intravesical ATP treatment are blocked in P2X_2_^–/–^ and P2X_3_^–/–^ mice. (**a**) Representative cystometrograms after 10 mM ATP instillation in wild-type, P2X_2_^–/–^, and P2X_3_^–/–^ mice. Scale bar, 10 min. (**b**) Changes in ICIs after ATP treatment (wild-type, P2X_2_^–/–^, *n* = 4 mice; P2X_3_^–/–^, *n* = 3 mice); **p = 0.0066 in wild-type mice (paired *t*-test with Holm correction) and ^†^*p* = 0.012 and 0.018 in wild-type versus P2X_2_^–/–^ and P2X_3_^–/–^ mice, respectively (time by group interaction using two-way repeated measures ANOVA with Holm correction). (**c**) Immunohistochemical analysis of c-Fos expression in L6–S1 spinal cord after ATP instillation in wild-type, P2X_2_^–/–^, and P2X_3_^–/–^ mice. Scale bars, 100 μm. (**d**) The number of c-Fos-positive cells in L6–S1 spinal cord after ATP instillation in wild-type, P2X_2_^–/–^, and P2X_3_^–/–^ mice (wild-type, P2X_2_^–/–^, *n* = 4 mice; P2X_3_^–/–^, *n* = 3 mice: 10 sections per mouse were assessed); **p = 0.0046 and 0.0023 in wild-type versus P2X_2_^–/–^ and P2X_3_^–/–^ mice, respectively (Tukey’s test following one-way ANOVA). Error bars represent s.e.m.

**Figure 8:**
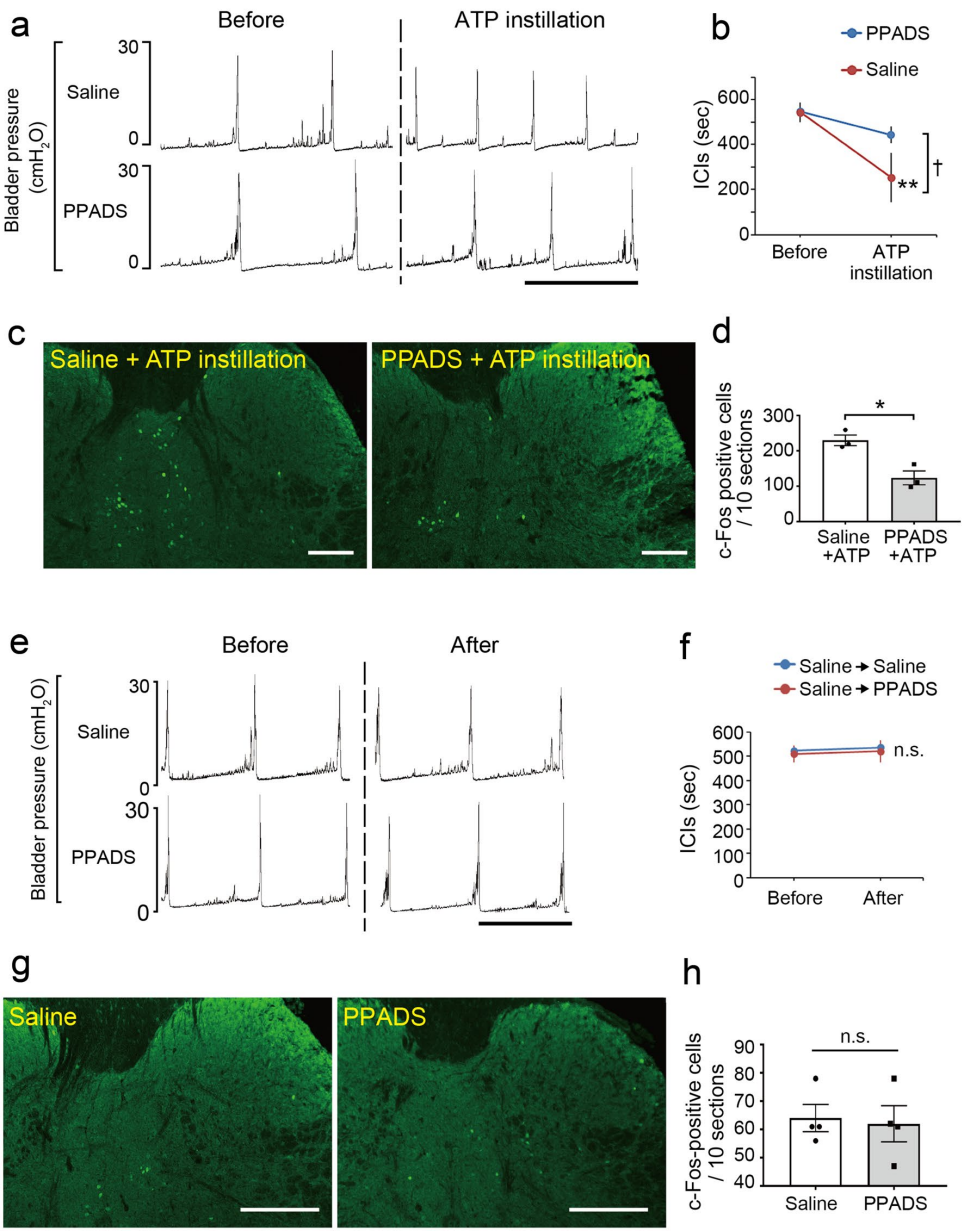
Effects of intravesical ATP treatment are blocked by PPADS, a non-selective purinergic receptor antagonist. (**a**) Representative cystometrograms after ATP instillation (20 mM) with or without PPADS treatment in wild-type mice. Scale bar, 10 min. (**b**) Changes in ICIs after ATP instillation with or without PPADS treatment (PPADS: *n* = 3 mice, Saline: *n* = 5 mice); **p = 0.0071 (paired *t*-test with Holm correction) and ^†^*p* = 0.026 (time by group interaction using two-way repeated measures ANOVA). (**c**) Immunohistochemical analysis of c-Fos expression in L6–S1 spinal cord after ATP instillation with or without PPADS treatment in wild-type mice. Scale bars, 100 μm. (**d**) The number of c-Fos-positive cells in L6–S1 spinal cord after ATP instillation with or without PPADS treatment (*n* = 3 mice per group: 10 sections per mouse were assessed); *p = 0.012 (Student’s *t*-test). (**e**) Representative cystometrograms after PPADS treatment in wild-type mice. Scale bar, 10 min. (**f**) Changes in ICIs after PPADS treatment (*n* = 4 mice, per group); *p* = 0.20 in saline and *p* = 0.38 in PPADS (paired *t*-test with Holm correction). (**g**) Immunohistochemical analysis of c-Fos expression in L6–S1 spinal cord after intravesical PPADS treatment in wild-type mice. Scale bars, 100 μm. (**h**) The number of c-Fos-positive cells in L6–S1 spinal cord after intravesical PPADS treatment (*n* = 4 mice per group: 10 sections per mouse were assessed); *p* = 0.81 (Student’s *t*-test). Error bars represent s.e.m., n.s., not significant.

The reduced number of ICIs (Fig. 7a,b), and the elevated number of c-Fos-positive cells in the L6–S1 spinal cord (Fig. 7c,d) following intravesical ATP instillation were significantly lower in P2X_2_^–/–^ and P2X_3_^–/–^ mice compared with wild-type mice (Fig. 7a–d) (Fig. 7b, ICIs: P2X_2_^–/–^ mice, ^†^*p* = 0.012; P2X_3_^–/–^ mice, ^†^*p* = 0.018, time by group interaction using two-way repeated measures ANOVA with Holm correction; Fig. 7d,c-Fos-positive cells: P2X_2_^–/–^ mice, **p = 0.0046; P2X_3_^–/–^ mice, **p = 0.0023, Tukey’s test following one-way ANOVA). In addition, intravesical treatment of wild-type mice with a non-selective purinergic receptor antagonist, pyridoxal phosphate-6-azophenyl-2,4-disulfonic acid (PPADS), inhibited the reduction in ICIs (Fig. 8a,b) (^†^*p* = 0.026, time by group interaction using two-way repeated measures ANOVA) and the increase in c-Fos-positive cell number in the L6–S1 spinal cord (Fig. 8c,d) (*p = 0.012, Student’s *t*-test), both of which were induced by intravesical ATP instillation. Treatment with PPADS alone did not affect ICIs (Fig. 8e,f) (*p* = 0.38, paired *t*-test with Holm correction) or the number of c-Fos-positive cells in the L6–S1 spinal cord (Fig. 8g,h) (*p* = 0.81, Student’s *t*-test). These results indicate that intravesical ATP treatment triggers activation of L6–S1 spinal neurons and induces an enhanced micturition reflex via purinergic receptors.

## Discussion

Recent studies of bacterial cystitis have focused on the immune response in the bladder^3,9–11,37,38^. These studies have clarified sophisticated defensive mechanisms induced by the innate immune system against bacterial cystitis^3,9–11^. Regarding the elimination of bacteria, urinary frequency, which is a common symptom of bacterial cystitis, appears to be an effective response against bacterial infection because micturition can rapidly expel bacteria from the bladder lumen to the outside of the body^34,35^. However, the mechanism underlying urinary frequency in bacterial cystitis has remained unclear. In this study, we revealed that TLR4 is strongly expressed on the luminal surface of the umbrella urothelium in the bladder, indicating that TLR4 comes into contact with urine and may, therefore, play a key role in the detection of luminal bacteria. Furthermore, our results show that LPS, a component of the bacterial cell wall, acts on TLR4 in the urothelium and induces ATP release from the urothelium. Moreover, our data indicate that ATP signaling via purinergic P2X_2_ and P2X_3_ receptors plays a pivotal role in the LPS-induced activation of L6–S1 spinal neurons through the bladder afferent pathway, resulting in rapid onset of an enhanced micturition reflex. We also showed that intravesical ATP treatment triggers activation of L6–S1 spinal neurons and enhances the micturition reflex. Thus, urinary frequency in bacterial cystitis is triggered by the bladder urothelium via an ATP-mediated pathway.

Interestingly, cystometry analysis of bladder function showed urinary frequency at 1 h after intravesical LPS instillation, when there were no inflammatory changes (e.g., neutrophil influx) in bladder tissue or urine (Fig. 1). These results indicate that the enhanced micturition reflex induced by ATP signaling is active prior to bacterial clearance by neutrophilic phagocytosis, which is induced by a conventional innate immune response in the bladder^3,9–11^. As urinary frequency can expel luminal extracellular bacteria without urothelial damage, increased bladder activity at the early stage of bacterial entry is quite effective. This agility of the bladder is a reasonable defense against bacterial infection and the rapid nerve conduction of this mechanism enables this rapid response.

The bladder urothelium functions as a physical barrier^6,7,49,50^ and a mechanosensor^51,52^ to maintain homeostasis. Furthermore, in this study, we identified a novel mechanism of the bladder urothelium in the defense against bacterial infection, mediated via neural signaling. Our results revealed that the bladder urothelium detects luminal extracellular bacterial LPS through TLR4 and converts the bacterial LPS information into neural signaling via an ATP-mediated pathway, resulting in urinary frequency. That is, the bladder urothelium plays an essential role as a chemical-neural transducer that converts bacterial LPS information into neural signaling via ATP. Our results, combined with those of previous reports, indicate that there are many similarities between bladder urothelial cells and sensory cells. Bladder umbrella cells detect a complex chemical substance, LPS, and then transmit that information to afferent nerve fibers through transmitter release. Thus, bladder urothelial cells act as sensory receptor cells for the detection of bacterial LPS. As the bladder lumen is directly open to the outside of the body and often faces invasion by foreign substances, the presence of such a mechanism is not surprising.

TLRs play a critical role in immune systems and are expressed on immunocompetent cells, such as monocytes, macrophages, and dendritic cells^53–55^. Recent studies have shown that non-immune cells also express TLRs, although the function of this non-immune localization remains to be fully elucidated^10^. This is the first report to show that TLR4 on the bladder urothelium couples neural signaling to the defense against bacterial infection, resulting in urinary frequency.

In the ATP release assay, we measured the ATP released from the mouse bladder urothelium towards the mucosal side after LPS treatment (Fig. 4). Furthermore, in the bladder cystometry and immunohistochemical analysis of the spinal cord, ATP was administered to the luminal side of the urothelium, which activates L6–S1 spinal neurons through the bladder afferents and enhances the micturition reflex (Figs. 6, 7, 8). Additionally, previous studies using an in vitro mouse/rat bladder-pelvic nerve preparation reported that intravesical administration of P2X receptor agonists, α,β-meATP or ATP, increased bladder afferent nerve firing^27,28,56^. Our results and previous reports indicate that ATP exerts its action on bladder afferents. However, because of an extraordinarily low permeability of the bladder urothelium, luminal ATP cannot cross this barrier easily and cannot activate the bladder afferent nerves directly^57,58^. How, then, does luminal ATP trigger activation of bladder afferent nerves? Previous studies revealed that purinergic receptors are expressed on urothelial cells, as well as on bladder afferent nerves^5,22,47^. In addition, extracellular ATP can induce its own release from urothelial cells via purinergic receptors^29,59^. Furthermore, ATP can be released from not only the apical but also the basolateral urothelial surface in response to stimuli^22^. These findings indicate that luminal ATP might act on purinergic receptors on urothelial cells, which then release ATP towards the basolateral side, which activates bladder afferent fibers located in close apposition to, and some within, the urothelium^7^ (Supplementary Fig. S4). Another possibility is that ATP could have an indirect action via the release of other transmitters, such as nitric oxide^60^ and acetylcholine^5^, from the urothelium towards the basolateral side, which in turn triggers afferent nerve activation (Supplementary Fig. S4). Thus, signals are transmitted from the luminal space to the underlying tissues via transmitter release from the urothelium to regulate bladder afferent nerves. This pathway is called ‘transmural signaling’^5^. The action of ATP via purinergic receptors could play a key role in transmural signaling, but further studies are required to clarify these possible mechanisms (Supplementary Fig. S4).

Wang et al. demonstrated ATP release from the mucosal and serosal surface of the rabbit bladder urothelium when exposed to increased hydrostatic pressure^22^. To measure ATP release towards the serosal side in an excised bladder, the smooth muscle layers overlaying the mucosal layer should be removed by dissection before mounting in an Ussing chamber. It is important to note, however, that the surgical action of removing the smooth muscle layers can damage the bladder tissue, initially increasing ATP release and, subsequently, decreasing ATP release as dead cells accumulate^23^. Therefore, this tissue preparation may affect the ATP concentration in the chamber solution. This is problematic for the ATP release assay. We attempted this tissue preparation with mouse bladders; however, it is difficult to remove the muscle layer without causing tissue damage. Although we could not assess ATP released towards the serosal side in the present analysis using an Ussing chamber, it is possible that LPS might induce ATP release from the serosal surface of the urothelium and then released ATP could directly activate bladder afferent fibers expressing P2X_2_ and/or P2X_3_ receptors^20,25,27^ (Supplementary Fig. S4). This hypothesis should be carefully examined in future studies.

Human^61–64^ and mouse^65^ urinary ATP levels are in the nM range. However, in previous studies that tested the effects of ATP given intravesically on mouse/rat bladder function, ATP concentrations at mM levels (10–60 mM) were used^43,57,58^. ATP is rapidly degraded by ecto-ATPase in the extracellular space of the bladder urothelium^58^. Additionally, a layer of polysaccharide, glycosaminoglycan and mucin, covers the superficial urothelium^7^. Therefore, it is speculated that ATP levels close to the purinergic receptors on urothelial cells are substantially lower than intravesically-administered ATP levels. In fact, previous studies reported that relatively high concentrations of ATP were needed to induce increased bladder activity by intravesical administration^57,58^. The ATP concentrations used in our experiments were as high as those in previous studies and could cause non-specific effects. However, the decreased ICIs in cystometry and the increased number of c-Fos-positive cells in the L6–S1 spinal cord following intravesical ATP administration were significantly prevented in P2X_2_^–/–^ and P2X_3_^–/–^ mice (Fig. 7), and by PPADS (a non-selective purinergic receptor antagonist) treatment (Fig. 8). Therefore, our present results induced by ATP administration can be accounted for by the specific involvement of purinergic receptors.

Bladder afferent nerves project to regions of the dorsal commissure (DCM), the superficial dorsal horn [medial dorsal horn (MDH) and lateral dorsal horn (LDH)], and the sacral parasympathetic nucleus (SPN)^17^. Previous studies analyzed neuronal c-Fos expression to examine the spinal processing of afferent inputs from the bladder, and showed that the distribution of c-Fos-positive cells in the spinal cord varied according to the stimulus^41,42^. Using rat bladders, Birder et al. showed that bladder distension increased c-Fos expression predominantly in the DCM and SPN regions^42^. Meanwhile intravesical chemical irritation with 1% acetic acid increased the number of c-Fos-positive cells in the DCM, SPN, and MDH regions, but not in the LDH region, compared with saline infusion^42^. In the present study using mouse bladders, intravesical LPS or ATP instillation induced an increase in the number of c-Fos-positive cells in the DCM and SPN regions, compared with saline instillation (Fig. 6g, Supplementary Figs. S2, S3). These results and results from others show that spinal neurons in the DCM and SPN regions have an important role in processing afferent input from the bladder in response to any stimulus. The pattern of involvement of neurons located in other regions seems to vary according to species and stimuli. It is also speculated that different stimuli might activate different types of afferents projecting to discrete regions of the spinal cord^42^. In addition, the failure to detect increased c-Fos expression does not necessarily mean that neurons have not been activated^41^. That is, some neurons may produce levels of c-Fos protein below the detection threshold of our immunohistochemistry. Thus, it is thought that the stimulus modality, species difference, and technical reasons may account for the varied distribution of c-Fos expression; however, further research is needed to clarify this interesting question.

In conclusion, we revealed a novel defensive mechanism against bacterial infection via an epithelial-neural interaction that induces urinary frequency. The umbrella urothelium senses luminal extracellular bacterial LPS in the bladder and releases the transmitter ATP, which triggers activation of neural circuits of the micturition reflex, resulting in the flushing out of bacteria. This mechanism is effective because it occurs more rapidly than bacterial clearance by neutrophilic phagocytosis induced by the innate immune system. Thus, the bladder urothelium plays an important role as a chemical-neural transducer that converts bacterial information into neural signaling via the transmitter ATP with bladder urothelial cells acting as sensory receptor cells. This sophisticated function of the bladder urothelium enables a quick and effective defensive response for maintaining homeostasis.

## Materials and methods

### Animals

Most experiments were conducted with 12–19-week-old male C57BL/6J, Purinergic Receptor P2X 2 knockout (P2X_2_^–/–^) (RRID: IMSR_JAX:004603), and Purinergic Receptor P2X 3 knockout (P2X_3_^–/–^) (RRID: IMSR_JAX:003951) mice. P2X_2_^–/–^ and P2X_3_^–/–^ mice^25,32^ on the C57BL/6J background were obtained from the Jackson Laboratory (Bar Harbor, ME, USA). C57BL/6 J mice were obtained from SLC (Shizuoka, Japan). The analysis of bladder tissue and urine after intravesical saline or lipopolysaccharide (LPS) exposure was conducted with 8–10-week-old female mice. Before all experiments, mice were housed under a 12 h:12 h light–dark cycle with controlled temperature and humidity and with food and water available ad libitum. All procedures were approved by the Institutional Animal Care and Use Committee of Osaka University and were conducted in accordance with EU Directive 2010/63/EU for animal experiments.

### Analysis of bladder tissue and urine after intravesical LPS exposure

The analyses of bladder tissue and urine after intravesical LPS exposure were conducted as previously described^37^. In brief, mice were anesthetized with 1.2 g/kg intraperitoneal urethane and placed in the supine position. A polypropylene 24-gauge catheter was inserted slowly via the urethra until backward flow of urine was visible. After drainage of all urine from the bladder, 150 μl saline or LPS from *Escherichia coli* 0111:B4, purified by phenol extraction (1.0 mg/ml) (Sigma, St Louis, MO, USA) was instilled^37,39,66,67^. To ensure consistent exposure of the bladder urothelium to saline or LPS, the syringe was maintained on the catheter for 1 h^37^. For histological analysis with hematoxylin and eosin staining, mice were deeply anesthetized with pentobarbital and the bladder was rapidly removed. For urine analysis, the urine in the bladder was collected with a syringe when the catheter was removed, and Samson staining was performed. For the 12 h LPS group, 150 μl saline was instilled 1 h before urine collection, as described above.

### Immunohistochemistry of the bladder urothelium

Mice were deeply anesthetized with pentobarbital and perfused with 1 × phosphate-buffered saline (PBS: pH 7.4) followed by 4% paraformaldehyde, as previously described^68^. The bladder was dissected, cryoprotected in 30% sucrose, rapidly frozen and then 10-μm sections were cut and mounted on slides. For immunohistochemical staining of Toll-like receptor 4 (TLR4), air-dried slides were rinsed with PBS and then treated in blocking solution of PBS containing 5% bovine serum albumin (Sigma) and 0.3% Triton X-100 for 1 h at room temperature (RT)^69^. Subsequently, the slides were incubated overnight at 4 °C with a polyclonal rabbit anti-TLR4 primary antibody (Abcam, Cambridge, UK; 1:400, Cat#ab13556, RRID: AB_300457) in blocking solution^70^. After washing with PBS, the slides were incubated with biotin-conjugated goat anti-rabbit IgG secondary antibody (Vector Laboratories, Burlingame, CA, USA; 1:200, Cat#BA-1000, RRID: AB_2313606) in PBS for 30 min at RT, followed by incubation in 0.3% H_2_O_2_ for 30 min at RT to eliminate endogenous peroxidases. Amplification with an avidin–biotin complex (ABC kit; Vector Laboratories) was performed and then reaction products were visualized with 50 mM Tris-buffered saline (TBS: pH 7.4) containing 1.25% 3′-diaminobenzidine and 0.75% H_2_O_2_. After stopping the reaction by immersion in 50 mM TBS, the slides were stained with hematoxylin (Merck KGaA, Darmstadt, Germany) as a counterstain. Bladders of TLR4^–/–^ mice (RRID: IMSR_OBS:4) were obtained from Oriental Bio Service (Kyoto, Japan)^71^. For hematoxylin and eosin staining, wild-type mice were deeply anesthetized with pentobarbital and the bladder was dissected, then placed in 4% paraformaldehyde. Subsequently, hematoxylin and eosin staining was performed. All slides were analyzed with a BX53 light microscope (Olympus Corporation, Tokyo, Japan).

### ATP release assay using an Ussing chamber

The analysis of ATP release from the bladder urothelium was performed as described previously^24^. In brief, the bladder was dissected, opened vertically, and sandwiched between the two halves of an Ussing chamber to act as a 7 mm^2^ diaphragm. The chamber volume of the mucosal side was 700 μl. Chambers were filled with Krebs solution (117 mM NaCl, 5.9 mM KCl, 1.2 mM MgCl_2_, 24.8 mM NaHCO_3_, 1.2 mM NaH_2_PO_4_, and 11.1 mM glucose), containing 2.5 mM CaCl_2_ and bubbled with 95% O_2_/5% CO_2_. LPS from *E. coli* 0111:B4, purified by ion-exchange chromatography (Sigma), was diluted to 1.0 mg/ml^24^. The TLR4 antagonist, TAK-242 (Calbiochem, La Jolla, CA, USA), was diluted to 1.5 mg/ml^72^ in Krebs solution. These solutions were added to the mucosal chamber as indicated. ATP concentration was measured in 50-μl samples from the mucosal chamber using a luciferin-luciferase method in accordance with the manufacturer’s protocol (Kikkoman, Chiba, Japan)^24^. ATP release was calculated by subtracting the pre-stimulation ATP concentration from the post-stimulation ATP concentration. The concentration of ATP was determined with calibration curves, which were constructed for each experiment using 3 × 10^−7^, 3 × 10^−8^, 3 × 10^–9^, and 3 × 10^–10^ M standard ATP solutions^24^.

### Cystometry

Cystometry was performed on mice using previously published methods^24,43,48^. In brief, mice were anesthetized with 1.2 g/kg intraperitoneal urethane and placed in the supine position. The bladder was exposed by a lower abdominal midline incision and a polyethylene catheter (PE-50; BD Japan, Tokyo, Japan) was inserted into the anterior wall of the bladder. The catheter was connected to an infusion pump (TE-351; Terumo, Tokyo, Japan) and a pressure transducer (DX-360; Nihon Kohden, Tokyo, Japan) for the measurement of intravesical pressure^24,43,48^. At first, the bladder was continuously infused with saline at 1.5 ml/h. After observation and analysis of stable baseline intercontraction intervals (ICIs), 1.0 mg/ml LPS from *Escherichia coli* 0111:B4, purified by phenol extraction (Sigma), was infused continuously into the bladder at a rate of 1.5 ml/h^24,48^. LPS infusion was continued for approximately 1 h and ICIs were evaluated. In ATP studies, ATP disodium salt hydrate (Tokyo Chemical Industry, Tokyo, Japan) was diluted to 5 mM, 10 mM, 20 mM, 50 mM, and 100 mM. These ATP solutions were adjusted to approximately pH 6.8 with NaOH solution to exclude the influence of acid-sensitive receptor activation or urothelial damage^43^. In male P2X_2_^–/–^ and P2X_3_^–/–^ mice, ICIs were analyzed with 10 mM ATP instillation. In antagonist studies, pyridoxal phosphate-6-azo (benzene-2, 4-disulfonic acid) tetrasodium salt hydrate (PPADS; Sigma), which is a non-selective purinergic receptor antagonist, was used. Wild-type mice were instilled with PPADS diluted to 1 mM^24^ for 3 h followed by instillation of PPADS (1 mM) and ATP (20 mM). ICIs after ATP or PPADS instillation were evaluated at approximately 1 h after treatments. In the TLR4 antagonist study, TAK-242 (Calbiochem) (1.5 mg/ml)^72^ was instilled in wild-type mice for 1 h followed by instillation of TAK-242 (1.5 mg/ml) and LPS (1.0 mg/ml). ICIs were evaluated at approximately 1 h after LPS treatment. Intravesical bladder pressures were recorded using Power Lab and Chart 5 (AD Instruments, Castle Hill, Australia).

### Bladder ultrasonography

Bladder ultrasonography was performed on mice using previously published methods^24,43,48^. In brief, mice were anesthetized with 1.2 g/kg intraperitoneal urethane and placed in the supine position. The ultrasonography probe was delicately placed on the abdomen with sonographic jelly. The maximum sagittal cross-sectional area (CSA) of the bladder was measured just before micturition, and the minimum CSA of the bladder was measured just after micturition by ultrasonography. The largest CSA and smallest CSA reflect maximum bladder capacity and post-voiding residual volume, respectively^24,43,48^. Bladder ultrasonography was performed with a Vevo 770 Imaging System equipped with a 25 MHz transducer (Visual Sonics, Toronto, ON, Canada) with saline or ATP instillation.

### Immunohistochemical analysis of c-Fos in L6–S1 spinal cord

Two hours after cystometry analysis, mice were deeply anesthetized with intraperitoneal pentobarbital and perfused with PBS followed by 4% paraformaldehyde^43,68^. Intravesical instillation of saline, LPS, and ATP was stopped just before pentobarbital injection. The L6–S1 segment of the spinal cord was carefully dissected and cryoprotected in 30% sucrose then rapidly frozen and cut transversely into 10-μm sections; one in every 10 consecutive sections was slide mounted. Air-dried slides were rinsed with PBS and blocked with 5% bovine serum albumin (Sigma) in PBS containing 0.3% Triton X-100 for 20 min at RT^69^. Subsequently, the slides were incubated overnight at 4 °C with a polyclonal rabbit anti-c-Fos primary antibody (Santa Cruz Biotechnology, Dallas, TX, USA; 1:1000, Cat#sc-52, RRID:AB_2106783) in blocking solution. After washing with PBS, the slides were incubated with a goat anti-rabbit Alexa 488-labeled secondary antibody (Invitrogen; 1:500, A11034) for 90 min at RT^70^. All slides were analyzed with a BX53 light microscope (Olympus Corporation). Ten consecutive sections from each mouse were selected for analysis and the numbers of c-Fos-positive cells in these sections were counted^43^. Detailed analysis of the distribution of c-Fos-positive cells was performed as previously described^41,42,45^. We divided the L6–S1 spinal cord into four regions: medial dorsal horn (MDH), lateral dorsal horn (LDH), dorsal commissure (DCM), and sacral parasympathetic nucleus (SPN), as previously described^41,42,45^. Ten consecutive sections of each mouse were analyzed and the number of c-Fos-positive cells in each region was counted.

### Statistical analysis

Animals were randomly assigned to treatment groups. For analysis of the ATP release assay, groups were compared using Tukey’s test following one-way analysis of variance (ANOVA) (Fig. 4) and one-way ANOVA (Supplementary Fig. S1). For immunohistochemical analyses of c-Fos-positive cells, Student’s *t*-test (Fig. 8d,h), Student’s *t*-test with Holm correction (Figs. 5d, 6g, Supplementary Fig. S2c) and Tukey’s test following one-way ANOVA (Figs. 5e, 6h, 7d, Supplementary Fig. S3) were performed. In addition, two-way repeated measures ANOVA with Holm correction was performed for analysis of LPS by group interaction for LPS instillation and in P2X knockout mice (Fig. 5d). For cystometry analysis of ICIs, paired *t*-test (Fig. 3d) and paired *t*-test with Holm correction (Figs. 3b, 5b, 6b, 7b, 8b, f) were performed. In addition, two-way repeated measures ANOVA (Figs. 3b, 8b) and two-way repeated measures ANOVA with Holm correction (Figs. 5b, 7b) were performed. For video-urodynamic analyses, Tukey’s test following one-way ANOVA was performed (Fig. 6d,e). All data are presented as means with standard errors of the means (s.e.m.) and values of *p* < 0.05 were considered statistically significant. All analyses were performed with JMP (SAS Institute, Cary, NC, USA).

## Supplementary information

Supplementary information.
