# Supplementary material for: Bladder urothelium converts bacterial lipopolysaccharide information into neural signaling via an ATP-mediated pathway to enhance the micturition reflex for rapid defense
Source: Sci Rep. 2020 Dec 3;10:21167. doi: 10.1038/s41598-020-78398-9 (PMC7713076; doi:10.1038/s41598-020-78398-9)
Supplement: Supplementary file 1 — Supplementary information. [file 41598_2020_78398_MOESM1_ESM.pdf]

Supplementary Information

Bladder urothelium converts bacterial lipopolysaccharide information into neural signaling via an ATP-mediated pathway to enhance the micturition reflex for rapid defense

Norichika Ueda †, Makoto Kondo †*, Kentaro Takezawa, Hiroshi Kiuchi, Yosuke Sekii, Yusuke Inagaki, Tetsuji Soda, Shinichiro Fukuhara, Kazutoshi Fujita, Motohide Uemura, Ryoichi Imamura, Yasushi Miyagawa, Norio Nonomura, Shoichi Shimada.

† These authors contributed equally to this work.
* Corresponding author: Makoto Kondo

Supplementary Figures and Legends

Supplementary Figure S1

Pre-stimulation ATP concentration in the ATP release assay.

Supplementary Figure S2

Intravesical LPS instillation increases the number of c-Fos-positive cells in the L6–S1 spinal cord.

Supplementary Figure S3

Intravesical ATP treatment increases the number of c-Fos-positive cells in the L6–S1 spinal cord.

Supplementary Figure S4

Schematic summary of hypothetical ATP signaling in bladder function.

Supplementary Figure S1

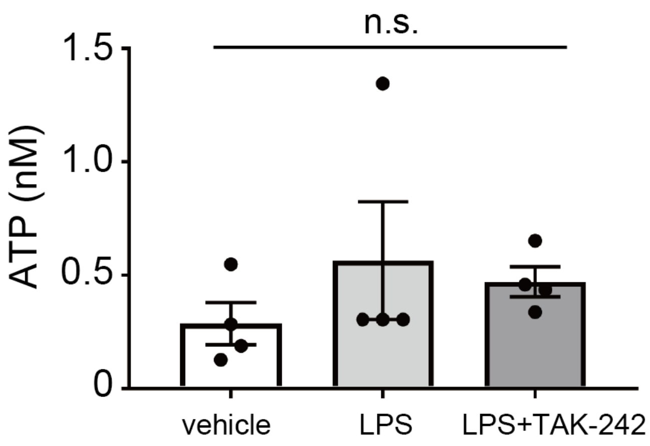

Supplementary Figure S1

Pre-stimulation ATP concentration in the ATP release assay.

There were no significant differences in the pre-stimulation ATP concentration among groups (n = 4 mice per group); $p = 0.50$ (one-way ANOVA). Error bars represent s.e.m., n.s., not significant.

Supplementary Figure S2

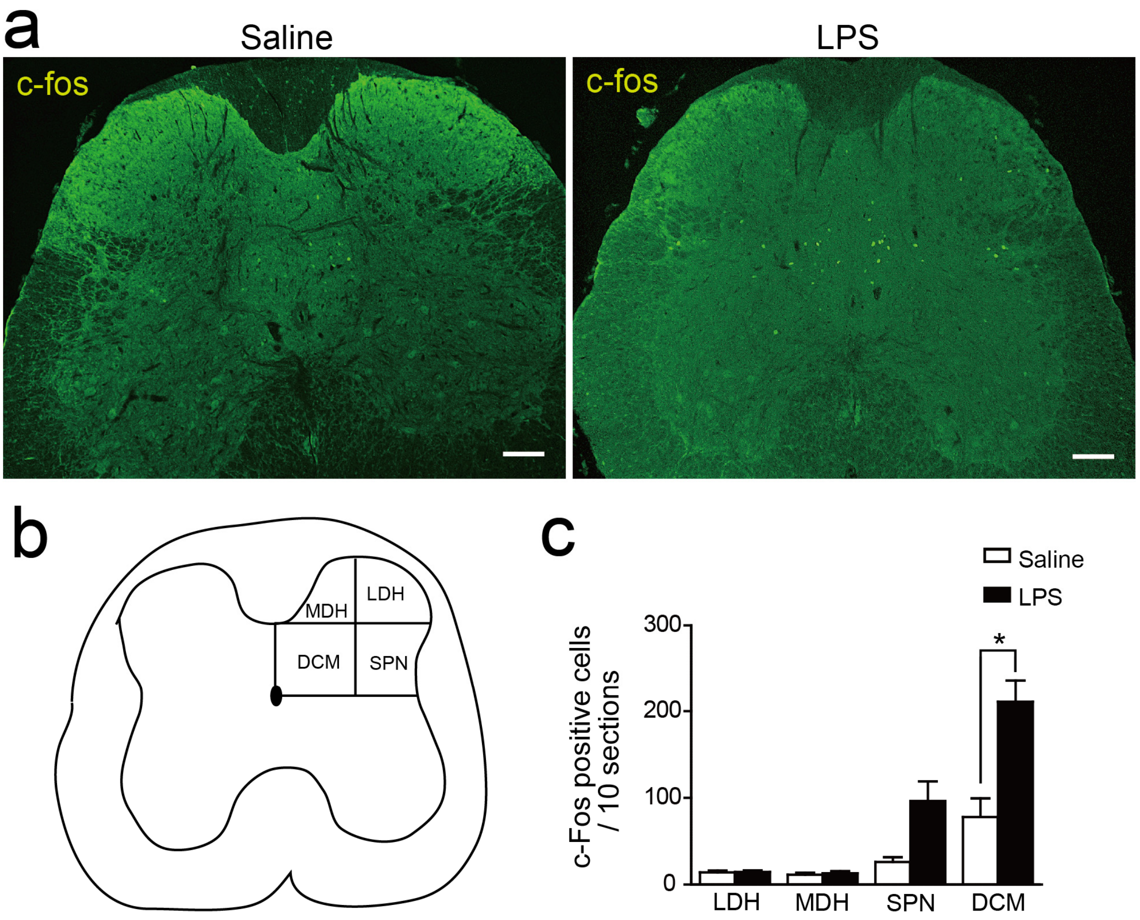

Supplementary Figure S2

Intravesical LPS instillation increases the number of c-Fos-positive cells in the L6–S1 spinal cord.

(a) Immunohistochemical images of c-Fos expression in L6–S1 spinal cord following saline and LPS instillation. Scale bars, 100 μm. **(b)** Schematic representation of four L6–S1 spinal cord regions; medial dorsal horn (MDH), lateral dorsal horn (LDH), dorsal commissure (DCM), and sacral parasympathetic nucleus (SPN). **(c)** The distribution of c-Fos-positive cells in the L6–S1 spinal cord induced by saline and LPS instillation (n = 4 mice per group: 10 sections per mouse were assessed); $^{*}p$ = 0.028 (Student's t-test with Holm correction). Error bars represent s.e.m.

Supplementary Figure S3

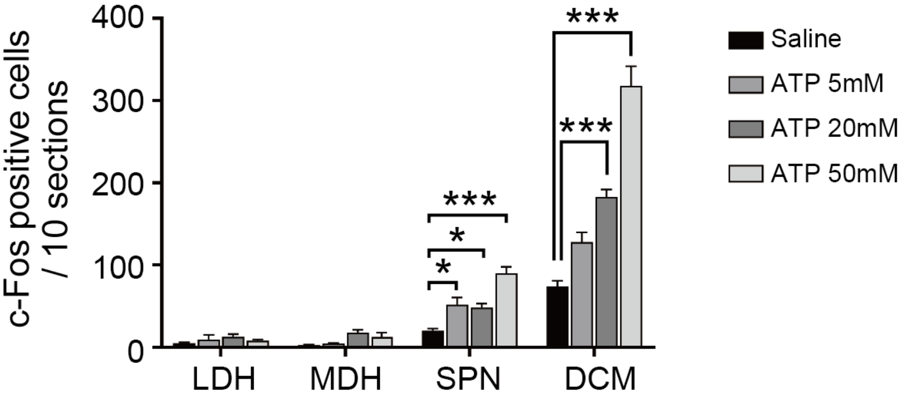

Supplementary Figure S3

Intravesical ATP treatment increases the number of c-Fos-positive cells in the L6–S1 spinal cord.

The distribution of c-Fos-positive cells in the L6–S1 spinal cord following saline and ATP instillation in wild-type mice (n = 5 mice per group: 10 sections per mouse were assessed); $^{*}p < 0.05$, $^{***}p < 0.001$ (Tukey's test following one-way ANOVA). Error bars represent s.e.m.

Supplementary Figure S4

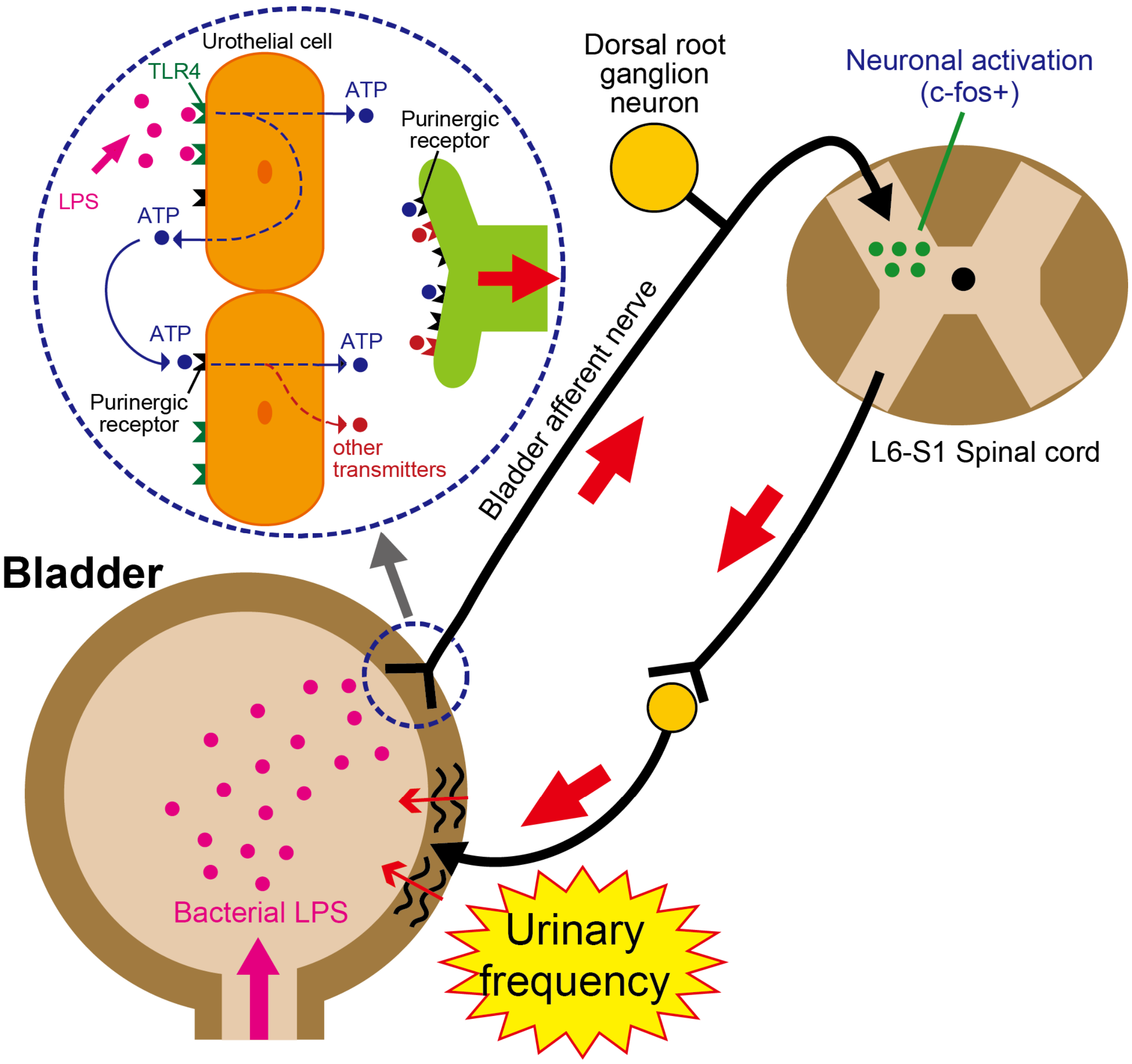

Supplementary Figure S4

Schematic summary of hypothetical ATP signaling in bladder function.

Bladder urothelium senses luminal bacterial LPS through TLR4 and releases the transmitter ATP. ATP signaling plays a pivotal role in the LPS-induced activation of L6–S1 spinal neurons through bladder afferent nerves, resulting in urinary frequency.
